# An Unorthodox Mechanism Underlying Voltage Sensitivity of TRPV1 Ion Channel

**DOI:** 10.1002/advs.202000575

**Published:** 2020-09-21

**Authors:** Fan Yang, Lizhen Xu, Bo Hyun Lee, Xian Xiao, Vladimir Yarov‐Yarovoy, Jie Zheng

**Affiliations:** ^1^ Department of Biophysics, and Kidney Disease Center of the First Affiliated Hospital Zhejiang University School of Medicine 866 Yuhangtang Road Hangzhou Zhejiang 310058 China; ^2^ Department of Physiology and Membrane Biology University of California, Davis One Shields Avenue Davis CA 95616 USA; ^3^ School of Life Sciences, Westlake Institute for Advanced Study Westlake University Shilongshan Road No. 18, Xihu District Hangzhou Zhejiang 310064 China

**Keywords:** ion channels, TRPV1, voltage gating

## Abstract

While the capsaicin receptor transient receptor potential vanilloid 1 (TRPV1) channel is a polymodal nociceptor for heat, capsaicin, and protons, the channel's responses to each of these stimuli are profoundly regulated by membrane potential, damping or even prohibiting its response at negative voltages and amplifying its response at positive voltages. Therefore, voltage sensitivity of TRPV1 is anticipated to play an important role in shaping pain responses. How voltage regulates TRPV1 activation remains unknown. Here, it is shown that voltage sensitivity does not originate from the S4 segment like classic voltage‐gated ion channels; instead, outer pore acidic residues directly partake in voltage‐sensitive activation, with their negative charges collectively constituting the observed gating charges. Outer pore gating‐charge movement is titratable by extracellular pH and is allosterically coupled to channel activation, likely by influencing the upper gate in the ion selectivity filter. Elucidating this unorthodox voltage‐gating process provides a mechanistic foundation for understanding TRPV1 polymodal gating and opens the door to novel approaches regulating channel activity for pain management.

## Graphical abstract

Voltage sensitivity of transient receptor potential vanilloid 1 (TRPV1) ion channel does not originate from the S4 segment like classic voltage‐gated ion channels; instead, outer pore acidic residues collectively constitute the observed gating charges. Outer pore gating‐charge movement is titratable by extracellular pH and is allosterically coupled to channel activation, likely by influencing the upper gate in the ion selectivity filter.

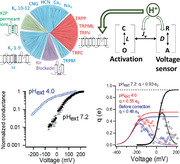

## 1 Introduction

When lipid bilayer emerged to enclose living cells more than three billion years ago,^[^ ^1^ ^]^ this diffusion barrier between cytoplasmic milieu and external environment allowed the establishment of transmembrane ion concentration gradients, which in turn yielded a transmembrane electric potential. Such a membrane potential (*V*_m_) has been widely utilized in cellular signaling:^[^ ^2^ ^]^ voltage‐gated ion channels alter *V*_m_ to elicit electrical signals for rapid communications;^[^ ^3^ ^]^ voltage‐sensitive enzymes such as Ci–VSP couple changes in *V*_m_ to the regulation of enzymatic activities and intracellular signaling.^[^ ^4^ ^]^ For these “classic” voltage‐sensing proteins, high sensitivity to voltage (fivefold per mV) has been attributed to a conserved, densely charged “voltage‐sensor” domain in the transmembrane region.^[^ ^5^ ^]^ Other membrane proteins—including G protein‐coupled receptors,^[^ ^6^ ^]^ ion channels and transporters^[^ ^7^ ^]^—have also evolved to take cues from *V*_m_ to perform their biological functions. Understanding the origin and operation of voltage sensitivity in these membrane proteins is of fundamental importance.

**Figure 1:**
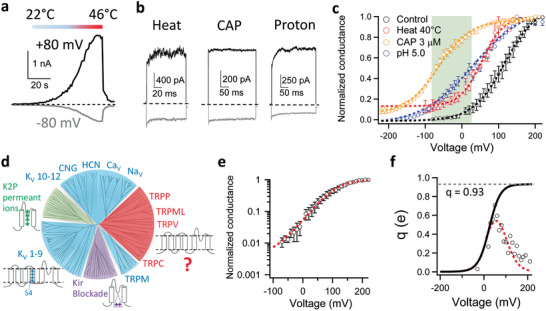
Voltage activation of TRPV1. a,b) Transmembrane voltage strongly modulates TRPV1 activities as shown in representative inside‐out patch recordings of a) TRPV1 activated by a heat ramp, b) high temperature at 42°C, 1 × 10^−6^ m capsaicin (CAP) or pH 5.0. Current traces recorded at depolarizing (+80 mV) and hyperpolarizing (−80 mV) voltages are in black and gray, respectively. c) Conductance–voltage (*G*–*V*) curve of TRPV1 (black: *V*_1/2_ = 114.9 ± 1.2 mV, apparent gating charge (*q*_app_) = 0.72 ± 0.06 e_0_, *n* = 4) was shifted to more hyperpolarized voltages in the presence of an activation stimulus (red, heat at 40°C: *V*_1/2_ = 54.8 ± 9.8 mV, *q*_app_ = 0.94 ± 0.20 e_0_, *n* = 3; Blue, extracellular pH 5.0: *V*_1/2_ = 33.4 ± 7.6 mV, *q*_app_ = 0.46 ± 0.03 e_0_, *n* = 4; Orange, CAP at 3 × 10^−6^ m: *V*_1/2_ = −72.3 ± 2.3 mV, *q*_app_ = 0.79 ± 0.19 e_0_, *n* = 4.). *G*–*V* curves are fitted to a single‐Boltzmann function (dash curves). Note that with CAP and heat, the *G*–*V* curves approach a steady‐state level around 0.1 at deeply hyperpolarized voltages. Shaded in green is the physiologically relevant membrane potential range, in which voltage exhibits a strong influence on channel activity. Conductances were normalized to the maximum conductance measured at the highest depolarization voltage. d) A rootless phylogenetic tree representing channels within the VGL ion channel superfamily. Sections in blue, green and purple cover channels with a known voltage‐sensing mechanism. Adapted with permission.^[^ ^13^ ^]^ Copyright 2004, The American Association for the Advancement of Science. e) Semilogarithmic plot of the voltage dependence of normalized mean conductance measured from steady‐state current in whole‐cell recordings. Then this plot was fitted with a smoothing function (dashed line in red) as described in Methods (Supporting Information with the parameters *V*_1/2_ = 107.99 mV, *q*_app_ = 0.72 e_0_ and base = 0.004.) This smoothing function (dashed line in red in Figure 1e) was then used to calculate *q*_a_ according to the Equation S3 in the Methods in Supporting Information. f) The voltage dependence of the derivative of mean log open probability. The *q*_a_ from the smoothing function was then plotted versus voltage in Figure 1f as the dashed line in red. Then the foot of *q*_a_(smoothing function)–*V* curve was fitted with Equation S4 in the Methods in Supporting Information to estimate the total gating charge, with the parameters *q*_0_ = 0.002 e_0_ and *q*_1_ = 0.928 e_0_. The total gating charge was estimated to be 0.93 e_0._ All statistics are given as mean ± s.e.m.

Transient receptor potential vanilloid 1 (TRPV1) channel, a polymodal nociceptor in higher species,^[^ ^8^ ^]^ is a good representative of the nonclassic voltage‐sensitive membrane proteins. TRPV1 is activated by noxious heat above 40°C,^[^ ^9^ ^]^ however, its response to heat at depolarized *V*_m_ is markedly larger than that at the resting *V*_m_ even when the driving force for its nonselective cation current is equal in magnitude (**Figure** **1**a). Likewise, TRPV1's responses to capsaicin and proton—which elicits spiciness sensation^[^ ^9^ ^]^ and the sustained phase of pain sensation,^[^ ^10^ ^]^ respectively—are also substantially asymmetrical (Figure 1b). Tuning of TRPV1's nociceptive sensitivity by *V*_m_ is a highly dynamic process occurring at human body temperature, with the range of voltage dependence (reflected by the conductance–voltage, or *G*–*V*, relationship) shifting over a more than 200 mV range in the presence of an activation stimulus^[^ ^11^ ^]^ (Figure 1c). Therefore, while TRPV1 is generally considered to be weakly voltage‐sensitive at room temperature, its activity is strongly influenced by membrane potential under physiological conditions (Figure 1c, shaded area). Dynamic voltage sensitivity makes TRPV1 more sensitive to noxious stimuli when its host sensory neuron has been previously excited (primed) or damaged, a general feature for nociception known as hyperalgesia.^[^ ^12^ ^]^ On the flip side, membrane hyperpolarization is expected to inhibit pain responses, offering opportunities for novel clinical intervention. Despite its physiological significance, how TRPV1 senses *V*_m_ remains largely elusive.

TRPV1 belongs to the voltage‐gated‐like (VGL) ion channel superfamily (Figure 1d),^[^ ^13^ ^]^ for which three voltage‐sensing mechanisms have been established. For voltage‐gated potassium (Kv), sodium (Nav), and calcium (Cav) channels (blue sections in Figure 1d), multiple regularly spaced basic residues on the transmembrane S4 helix enable it to move in response to *V*_m_ changes.^[^ ^14^ ^]^ For two‐pore domain potassium channels (K2P) without a voltage‐sensor domain (green section in Figure 1d), permeant cations have been proposed to bestow voltage sensitivity.^[^ ^15^ ^]^ For inward‐rectifier potassium channels (Kir) (purple section in Figure 1d), voltage‐dependent pore block by endogenous polyamines and Mg^2+^ ions introduces voltage sensitivity.^[^ ^16^ ^]^ These three mechanisms are applicable to about three‐fourth of the VGL superfamily; voltage‐sensing is less understood for a significant portion of the family (red section) that is composed of cellular sensor TRP channels including TRPV1. Our study began by determining whether TRPV1 employs one of these three known mechanisms or a new mechanism to detect *V*_m_.

## 2 Characterizing TRPV1's Voltage Sensitivity

A fundamental parameter for voltage sensitivity is the total charge movement, *q*, which determines the steepness of voltage response.^[^ ^5^ ^]^ TRPV1 is known to exhibit shallow voltage dependence.^[^ ^11^ ^]^ By fitting a Boltzmann function to the *G*–*V* curve of mouse TRPV1 recorded in HEK293 cells (Figure 1c), the apparent *q* was estimated to be 0.72 ± 0.05 e_0_ (*n* = 4). Applying this approach to Kv channels yielded an apparent *q* of 5.3 e_0_.^[^ ^17^ ^]^ However, it is well known that fitting the shape of *G*–*V* curves could yield an underestimate of *q*, for example, when there are multiple voltage‐dependent closed states that the channel may traverse before reaching the open state.^[^ ^17^, ^18^, ^19^ ^]^ Indeed, the total gating charge of Kv, K2P, and Kir channels are about 13‐to‐16 e_0_,^[^ ^18^, ^20^, ^21^, ^22^ ^]^ 2.2 e_0_^[^ ^15^ ^]^ and 2.2 e_0_,^[^ ^16^ ^]^ respectively.

The relatively weak voltage sensitivity of TRPV1 precluded direct measurement of *q* from gating current. Alternatively, the limiting‐slope method^[^ ^23^ ^]^ is a classic approach to estimate *q*, for which voltage dependence was measured at low open probabilities. However, the voltage‐driven transition of TRPV1 is allosterically coupled to activation gating,^[^ ^24^ ^]^ with the channel open probability approaching a stable level at deep hyperpolarization (Figure 1e) that reflected the equilibrium of activation gate between the closed and open state.^[^ ^25^ ^]^ To better estimate *q*, we first calculated the derivative of the mean log open probability with respect to voltage and fitted the Boltzmann smoothing function of voltage dependence of mean log open probability (see Equation S4 in Methods in the Supporting Information for details) (Figure 1f), as was previously performed on the allosteric large‐conductance calcium‐activated Potassium (BK) channels.^[^ ^25^ ^]^ This approach yielded an estimated *q* of 0.93 e_0_, which is 30% larger than that measured from fitting a Boltzmann function to the *G*–*V* curve (0.72 e_0_), yet much smaller than that of BK channels (2.62 e_0_).^[^ ^25^, ^26^ ^]^ To estimate the potential deviation of the gating charge estimate from its true value, we simulated the voltage sensitivity of TRPV1 open probability based on a simple allosteric scheme (Extended Data Figure S1a, Supporting Information). With the hypothetical gating charge value set to span a wide range from 1.0 e_0_ to 13.0 e_0_, our estimations (Extended Data Figure S1b–e, Supporting Information, dotted curves in red) were close to the true value, with deviations ranging from 10% to 13% (Extended Data Figure S1f, Supporting Information). When the method was applied to BK channels, for which total gating charge could be directly measured from the gating current, an underestimation of 7.7% was seen.^[^ ^25^, ^26^ ^]^ Therefore, the method we employed can reasonably estimate the gating charge.

## 3 TRPV1 S4 Does Not Serve as a Voltage Sensor

**Figure 2:**
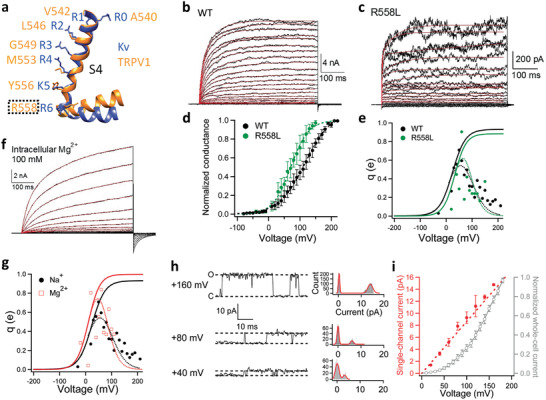
None of the three classic voltage‐sensing mechanisms appears applicable to TRPV1. a) Structural alignment of the S4 helixes in TRPV1 (Orange; PDB ID: 5IRZ) and Kv 1.2–2.1 chimera channel (Blue; PDB ID: 2R9R). b,c) Representative whole‐cell recordings of wild‐type (WT) TRPV1 and its mutant R558L activated by depolarization with 10 mV increments from −80 to +220 mV and from −40 to +150 mV, respectively. The voltage activation time courses are well fitted to a double‐exponential function (red traces). d,e) *G*–*V* curves of WT (black) and R558L (green, *V*_1/2_ = 71.4 ± 12.3 mV, *q*_app_ = 0.96 ± 0.06 e_0_, *n* = 3.) in linear plot. e) The voltage dependence of the derivative of mean log open probability of WT (black) and R558L (green). Dashed curve is a Boltzmann smoothing function determined from fits to mean log open probability. Solid curve is a fit of the smoothing function with a Boltzmann function at voltages where the open probability is low. f) A representative whole‐cell recording with Mg^2+^ as permeating ions. The voltage activation time courses are well fitted to a double‐exponential function (red traces). g) The voltage dependence of the derivative of mean log open probability of WT measured with intracellular sodium ions (black) and magnesium ions (blue). Dashed curve is a Boltzmann smoothing function determined from fits to mean log open probability. Solid curve is a fit of the smoothing function with a Boltzmann function at voltages where the open probability is low. h) Representative single‐channel recordings from an inside‐out patch clamped at +40, +80, and +160 mV, respectively. To determine the single‐channel current amplitude, their corresponding all‐point histograms are fitted to a double‐Gaussian function (curves in red). i) Voltage dependence of single‐channel current amplitude (red) and macroscopic current recorded in the whole‐cell configuration (gray) (*n* = 3–5). All statistics are given as mean ± s.e.m.

The S1–S4 domain of TRPV1 channels, like its counterpart in Kv channels,^[^ ^27^ ^]^ forms a compact domain surrounding the channel pore in a domain‐swapped arrangement.^[^ ^28^, ^29^ ^]^ In Kv channels, this domain serves as the main voltage sensor.^[^ ^20^ ^]^ To test whether TRPV1 also relies on the S1–S4 domain to sense *V*_m_, we first compared the S4 sequences (**Figure** **2**a). The Kv channel S4 segments contain seven regularly spaced charged residues. The corresponding residues in TRPV1 are all uncharged except for R558, which matches to R6 of Kv at the end of S4 thought to be insignificant for voltage‐sensing.^[^ ^20^ ^]^ To test for a potential contribution of R558 to voltage sensitivity, we mutated this residue to leucine. Charge‐neutralization did not abolish voltage activation (Figure 2b,c). While the *G*–*V* curve of R558L was left‐shifted (Figure 2d), the estimated *q* value, at 0.88 e_0_, was not significantly reduced compared to that of the wild‐type channels (Figure 2e). Similar observations were previously reported when the equivalent residue in rTRPV1 was mutated to A, L, and K.^[^ ^30^ ^]^ Therefore, R558 in TRPV1 S4 cannot serve as the main gating charge carrier. These results showed that the S4 voltage‐sensing mechanism of Kv channels is not applicable to TRPV1.

## 4 Permeant Ions Do Not Affect Voltage Sensitivity of TRPV1

Since an S4‐based voltage‐sensing mechanism is inapplicable to TRPV1, we examined other known mechanisms. To test whether TRPV1 uses permeant ions to sense voltage like K2P channels, we took advantage of TRPV1's low ion selectivity.^[^ ^8^ ^]^ We reasoned that, if *q* is originated from permeant ion movement, switching from monovalent cations to divalent cations would likely alter the measured *q* value, doubling it if these cations bind to the same site(s) in the pore. When the intracellular permeant ions were switched from Na^+^ to Mg^2+^, the voltage activation and deactivation kinetics were both much slower (Figure 2f). The *G*–*V* curve was right‐shifted but without any noticeable change in shape (Extended Data Figure S2, Supporting Information), with an estimated total gating charge of 0.99 e_0_ (Figure 2g). Therefore, unlike in K2P channels, permeant ions do not appear to serve as the voltage sensor for TRPV1.

## 5 TRPV1's Voltage Sensitivity Is Not Originated from Permeation Block

Like K2P channels, Kir channels also lack the S1–S4 voltage sensor domain. Their currents exhibit voltage‐dependence because of voltage‐dependent pore block by endogenous polyamines and Mg^2+^ ions at depolarized voltages.^[^ ^16^ ^]^ Such pore block is much faster than the resolution of patch‐clamp recordings so that, when it occurs, the single‐channel conductance is reduced. As a result, the single‐channel current amplitude exhibits similar voltage dependence as the macroscopic current.^[^ ^31^ ^]^ To test whether TRPV1 employs this pore‐block mechanism for sensing voltage, we compared single‐channel current and macroscopic current recorded at voltages where TRPV1 was clearly activated by depolarization (Figure 2h,i). It was obvious that the single‐channel current amplitude was linearly dependent on voltage and did not follow the voltage dependence of macroscopic current (Figure 2i). In contrast, the voltage dependence of single‐channel open probability was similar to the *G*–*V* curve measured from whole‐cell recordings (Extended Data Figure S3, Supporting Information), which is also consistent with a previous study.^[^ ^32^ ^]^ Moreover, in inside‐out patch recordings the voltage dependence did not decrease upon continuous perfusion of the bath solution for about three minutes (Extended Data Figure S4, Supporting Information). In comparison, Kir channels lost the inward‐rectification feature within two minutes of patch excision due to wash‐off of endogenous blockers.^[^ ^16^ ^]^ Therefore, unlike Kir channels, voltage sensitivity of TRPV1 is unlikely originated from pore block.

## 6 TRPV1 Voltage‐Dependent Gating Behavior Resembles a Concerted Transition

**Figure 3:**
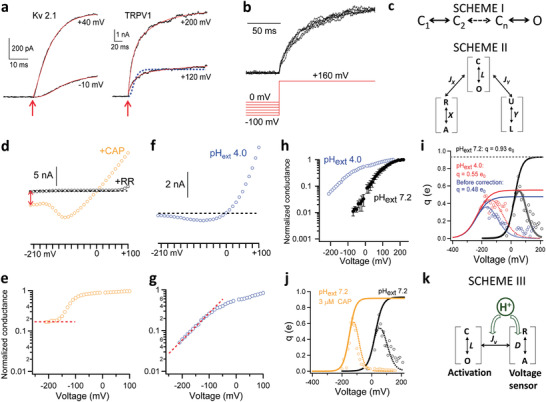
Voltage‐sensing of TRPV1 involves the proton activation machinery. a) Voltage activation kinetics of Kv 2.1 channels exhibit an initial delay between depolarization (marked by an arrow in red) and the onset of macroscopic current. No such a delay was observed in voltage activation of TRPV1. The activation time course of Kv 2.1 is fitted to Equation S2, Supporting Information (based on SCHEME I in (c); also see Methods in Supporting Information), whereas a double‐exponential function is used to fit that of TRPV1 (solid curves in red). SCHEME I predicted the activation time course of TRPV1 shown by the dashed trace in blue. b) No Cole‐Moore shift was observed in voltage activation of TRPV1. No delay in current onset can be observed from macroscopic currents recorded at +160 mV after a 2 s prepulse from 0 mV to −100 mV with 20 mV increments. c) Gating SCHEME I and II for voltage activation of Kv channels and TRPV1, respectively. d) A representative recording of the voltage dependence of TRPV1 in whole‐cell configuration in the presence of 10 × 10^−6^ m CAP. Orange circles represent the steady‐state current amplitude at the corresponding voltages. Leak current was measured from the same patch in the presence of ruthenium red (20 × 10^−6^ m) to block TRPV1 (black circles). Current through TRPV1 at −210 mV is indicated by a double‐arrow in red. A dashed line in black indicates the zero‐current level. e) Voltage dependence of the normalized conductance calculated from the recording in (d). A dashed line in red indicates a steady‐state level was reached at deeply hyperpolarized voltages beyond −160 mV. f) A representative recording of the voltage dependence of TRPV1 in whole‐cell configuration in the presence of extracellular pH 4.0. g) Voltage dependence of the normalized conductance calculated from the recording in (f). A dashed line in red indicates the conductance level kept decreasing at deeper hyperpolarization. h) The semilogarithmic plot for the voltage dependence of normalized mean conductance measured from steady‐state current in whole‐cell recordings at extracellular pH 4.0 and pH 7.2, respectively. i) The fits to the derivative of the mean log open probability at extracellular pH 4.0 and pH 7.2, respectively. For pH 4.0 condition, the averaged *q* values from three independent recordings were fitted, with the parameters *V*_1/2_ = −106 mV, *q*_app_ = 0.63 e_0_ and base = 0.04 for the dashed smoothing function and *q*_0_ = 0.0007 e_0_ and *q*_1_ = 0.55 e_0_ for the Boltzmann function to estimate the total gating charge (see Methods for details in Supporting Information). The total gating charge was estimated to be 0.55 e_0._. Dashed curve is a Boltzmann smoothing function determined from fits to mean log open probability. Solid curve is a fit of the smoothing function with a Boltzmann function at voltages where the open probability is low. All statistics are given as mean ± s.e.m. j) The fits to the derivative of the mean log open probability with and without 3 × 10^−6^ m capsaicin, respectively. k) A gating scheme where the voltage‐ and H^+^‐dependent transition couples allosterically to channel opening.

As none of the known voltage‐sensing mechanisms in the VGL channel superfamily is applicable to TRPV1, an unorthodox mechanism exists. We gained the first glimpse of such a mechanism from kinetic measurements. Voltage activation of Kv2.1 channel exhibited a sigmoidal time course^[^ ^22^ ^]^ (**Figure** **3**a), where there was an initial delay in macroscopic current upon depolarization (marked by red arrow). This delay is due to multiple closed states Kv channels must traverse before reaching the open state^[^ ^17^, ^33^ ^]^ (Figure 3c, SCHEME I). In contrast, voltage activation of TRPV1 nicely followed a double‐exponential time course (Figures 3a and 2b, red solid traces), with no detectable delay in current onset. Indeed, time courses predicted by gating schemes for Kv channel in the form of SCHEME I poorly described the voltage activation kinetics of TRPV1 (Figure 3a, blue dash trace). In addition, it is well established that for Kv channels holding *V*_m_ at more hyperpolarized voltages would produce a longer delay in current activation upon depolarization. Such a longer delay, named Cole‐Moore shift,^[^ ^34^ ^]^ is again due to the existence of multiple closed states preceding the open state. When TRPV1 channels were voltage‐clamped at various hyperpolarized voltages, there was no detectable Cole‐Moore shift (Figure 3b). The absence of both initial delay in macroscopic current activation and Cole‐Moore shift, as well as the double‐exponential current time course, can be explained by an allosteric model as shown in Figure 3c, SCHEME II, in which there is a single concerted voltage‐dependent transition that may represent either the C↔O transition or a separate transition that is allosterically coupled to the C↔O transition. (The observation of a double‐exponential activation time course rules out a simpler two‐state C↔O model for voltage‐dependent activation.) More complex systems, for example, one with multiple rapid voltage‐dependent transitions and a single rate‐limiting voltage‐independent transition right before the first opening transition, could also produce similar observations in channel activation time course and the absence of a Cole‐Moore shift.^[^ ^35^ ^]^ The simple allosteric model in Figure 3c, SCHEME II, is consistent with additional results described below.

## 7 Identifying the Voltage‐Dependent Transition

Besides membrane depolarization, TRPV1 can be allosterically activated by distinct physical and chemical stimuli, many of which have been better characterized both structurally and functionally.^[^ ^24^, ^36^ ^]^ A multiallosteric gating model initially proposed for BK channels^[^ ^37^ ^]^ can adequately describe TRPV1 activation.^[^ ^32^, ^38^, ^39^ ^]^ The polymodal nature of TRPV1 activation offered an opportunity to identify the voltage‐dependent transition. For a general allosteric system, there exist three possible scenarios as shown by SCHEME II (Figure 3c) that are experimentally distinguishable by measuring open probability, P_o_, at deeply hyperpolarized voltages:

a) Voltage directly controls the C↔O transition. In this scenario, the channel can be tightly held in the C state by hyperpolarization; no other stimuli should be able to evade voltage's control over the C↔O transition to cause channel activation.
2) Voltage controls a transition, R↔A, that is allosterically coupled to the C↔O transition, and another stimulus, *X*, also activates the channel through the same transition. In this scenario, hyperpolarization can effectively prevent channel activation by *X* but not by other stimuli (for example, *Y*).
3) Voltage controls a distinct transition, independent of other stimuli, that is allosterically coupled to the C↔O transition. In this scenario, all other stimuli can efficiently activate the channel at hyperpolarized voltages.

To distinguish these scenarios, we measured NP_o_ from single‐channel events at deep hyperpolarization down to −210 mV in the absence or presence of another stimulus. We found that, in the absence of another stimulus, NP_o_ appeared to approach a steady level at deeply negative voltages (Extended Data Figure S5, Supporting Information). In agreement with previous reports,^[^ ^32^, ^38^, ^40^, ^41^ ^]^ the observed baseline *P*_o_ level was very low, with an upper limit estimate near 0.01 at room temperature (Figure 1e). The presence of a voltage‐independent baseline *P*_o_ is inconsistent with Scenario A, suggesting that the C↔O transition itself must be associated with little charge movement and have an intrinsic open probability (*P*_o_intrinsic_) of less than 0.01.

In the presence of a saturating concentration (3 × 10^−6^ m) of capsaicin, TRPV1 current was readily detectable even at −210 mV (Figure 3d, red arrow). The current could be almost completely blocked by ruthenium red (Figure 3d, black circles), confirming that contamination by leak current must be small. The estimated *P*_o_ plateaued above 0.1 when voltage was hyperpolarized below −160 mV (Figure 3e, red dash line; see also Figure 1c). Similar to previous reports,^[^ ^32^, ^41^ ^]^ the presence of a stable *P*_o_ substantially above *P*_o_intrinsic_ indicated that capsaicin could allosterically promote the C↔O transition even at deep hyperpolarization. Therefore, voltage activation cannot be carried out through the same transition promoted by capsaicin, i.e., if Scenario B is true, capsaicin cannot be stimulus *X*. Indeed, as capsaicin^[^ ^42^, ^43^ ^]^ and other pungent natural compounds^[^ ^44^ ^]^ induce TRPV1 activation by linking S4 to the S4–S5 linker,^[^ ^45^ ^]^ these results offered further supports for the conclusion that voltage activation does not involve S4. Furthermore, the observation of a voltage‐independent, elevated P_o_ in the presence of capsaicin also validated the conclusion that voltage cannot work through the C↔O transition to open the channel, i.e., Scenario A is invalid.

Like capsaicin, we found that many other TRPV1 activation stimuli, including heat,^[^ ^46^ ^]^ were also effective at deeply hyperpolarized voltages (see, for example, Figure 1c). However, the situation was different when extracellular protons were used as the activator. We found that even in the presence of a saturating concentration of protons (at pH 4.0), current decreased towards zero at hyperpolarizing voltages (Figure 3f). No plateau could be observed in the *P*_o_–*V* plot (Figure 3g); instead, *P*_o_ kept decreasing towards *P*_o_intrinsic_ (Figure 3h). It is known that protons partially block TRPV1 permeation, which also exhibits a shallow voltage dependence.^[^ ^47^ ^]^ However, proton blockade had been corrected for before calculating *P*_o_ (Extended Data Figure S6, Supporting Information). Therefore, these observations fit the prediction of Scenario B in that deep hyperpolarization can shut down proton activation, identifying that voltage works though the proton activation pathway. These results also voided Scenario C by identifying protons as the stimulus *X*.

## 8 Location of the Gating Charges

While unanticipated, the finding that voltage sensitivity of TRPV1 resides in the proton activation pathway might not be surprising. It is well established that proton activation involves protonation of charged residues in the outer pore,^[^ ^48^ ^]^ a region intimately involved in TRPV1 activation gating and known to undergo substantial conformational rearrangements.^[^ ^49^ ^]^ The outer pore is also noticeably rich in charged residues (Extended Data Figure S7a, Supporting Information). If some of these charged residues locate within or near the *V*_m_ field, their movements during channel activation will impart voltage sensitivity to the process.^[^ ^5^ ^]^ Because the estimation of gating charge can be influenced by the value of *P*_o_, which increases at low pH, we also estimated *q* using a *Q*_a_/(1‐*P*_o_) correction (see Methods, Supporting Information). We observed that before correction the gating charge was reduced to 0.48 e_0_ by pH 4.0 (Figure 3i, circles and curves in blue); with the correction applied to voltage below 0 mV the gating charge was determined to be 0.55 e_0_ (circles and curves in red) which, though slightly larger than 0.48 e_0_, is substantially smaller than the gating charge measured at neutral pH. Given that the *p* *K*a value for the sidechain of negatively charged Glu or Asp is around 4, at extracellular pH 4.0 only about half of these charged residues would be neutralized. Therefore, we reasoned that though we observed only an around 50% reduction of *q* at acidic pH, nearly all of the 0.93 e_0_ total gating charge could be collectively contributed by titratable acidic residues.

Consistent with a previous report,^[^ ^50^ ^]^ no obvious change in the total charge movement could be confidently identified from channels carrying single or double charge neutralization mutations (Extended Data Figure S7b,c, Supporting Information), likely because their individual impacts to *q* were too small to stand out of the noise. These mutagenesis tests confirmed that the total gating charge of about 1 e_0_ is not carried by any single charged residue (which would require the residue to move nearly 1/4 of the whole *V*_m_ field during activation). Instead, it is most likely that multiple charged residues move collectively during voltage gating. As a negative control we observed no change in total gating charge in the presence of the classic TRPV1 agonist capsaicin (Figure 3j).

To test whether the reduction in apparent gating charge represented a decrease in total gating charge rather than a decrease in the coupling strength between voltage sensor and the gate (*J*_v_ in the allosteric gating model in Extended Data Figure S1a, Supporting Information), we have first performed the simulation of voltage activation at neutral pH with a simple allosteric gating model (Extended Data Figure S1a, Supporting Information) with the parameters shown in Extended Data Figure S8a (Supporting Information). We observed that with the set of parameters shown in blue, the simulated *P*_o_–*V* curve and *Q*_a_–*V* curve were similar to our experimentally measured voltage dependence of open probability at neutral pH (dotted curve in red). From the simulation, we observed that when *J*_v_ was reduced by 10‐fold, from 30 to 3, the apparent gating charge could be reduced by half (Extended Data Figure S8b, Supporting Information, dashed curve in black). However, the reduction in *J*_v_ led to an expected large decrease in the maximum *P*_o_ (dashed curve in black), which was inconsistent with our experimental measurements. In contrast, when we reduced the total gating charge (*z*) by half, the determined *Q*_a_ was reduced by half (Extended Data Figure S8b, Supporting Information, dashed curve in green) without a reduction in the maximum *P*_o_ (panel b, dashed curve in green). Therefore, we consider that the decrease in peak *Q*_a_ value is likely caused predominantly by a reduction in total gating charge, but not the coupling (*J*_v_).

Our gating charge estimates depend on fitting the mean log open probability with a smoothing function (Equation S4, Supporting Information) that includes a “base” term to describe the intrinsic *P*_o_ of the channel, which is difficult to measure accurately. Therefore, to test the influence of variations in the “base” term in the smoothing function (Equation S4, Supporting Information) on the total gating charge estimation, we reduced the base term by 10‐fold, from 0.04 to 0.004. We found that the *Q*_a_–*V* curves of a reduced base (Extended Data Figure S8c, Supporting Information, solid curves in blue and red for without or with *Q*_a_/(1‐*P*_o_) correction, respectively) were obviously inconsistent with the experimentally measured values (open circles). Nevertheless, we fitted the *Q*_a_–*V* curve and observed that the gating charge value would be 0.58 e_0_, which is only slightly larger (5.5%) than the 0.55 e_0_ value determined with the base term of 0.04. Therefore, the base term value of 0.04 is reasonable and our calculated gating charge value is robust against variations in the base term value.

To better simulate the voltage dependence of TRPV1 channel open probability, we constructed an allosteric gating model (Extended Data Figure S9a, Supporting Information) that includes voltage‐ and capsaicin‐dependent transitions. With this model, we found that a dedicated proton sensor was not required if protonation could decrease the total gating charge and strengthen coupling between voltage sensor and the activation gate (*J*_v_). With Equation S6, Supporting Information and the parameters listed in Extended Data Table S3 (Supporting Information), the predicted *P*_o_–*V* relationship of this allosteric gating model (solid curves in Extended Data Figure S9b, Supporting Information) was in reasonable agreement with our measured voltage dependence of TRPV1 open probability at neutral pH, low pH and with capsaicin (Extended Data Figure S9b, Supporting Information, open circles in blue, green, and red, respectively). While this allosteric gating model qualitatively describes our experimentally measured voltage dependence of TRPV1 open probabilities, we acknowledge that it is meant to illustrate the basic feature of coupling between voltage and protons. The model would be too simple to describe every details of experimental data (which is not the goal of this study). Nevertheless, this model (Scheme III; Figure 3k) indicates the proton and voltage activation mechanisms are deeply intertwined.

## 9 Structural Mechanism for Voltage/Proton Activation

**Figure 4:**
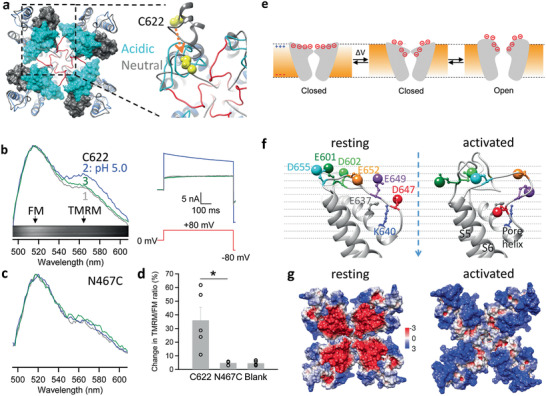
Structural mechanism underlying voltage sensitivity in TRPV1. a) The pore region of TRPV1 modeled under acidic (pH 4.0) and neutral conditions based on the cryo‐EM structure in the closed state (PDB ID: 3J5P). For the model under acidic pH, its selectivity filter and the linker to S6 are colored in red. The turrets under acidic and neutral pH are shown in cyan and dim gray, respectively. Atoms of C622 on the turret are shown as spheres in yellow. An arrow in orange indicates the direction of C622 movement upon acidification. b) Proton‐induced conformational changes at the turrets measured by whole‐cell patch fluorometry from FM and TMRM attached to C622 sites. 1, 2, and 3 indicate the emission spectra and their corresponding current recordings at neutral pH, perfusion of a pH 5.0 solution and wash off, respectively. The emission spectra were normalized to the emission peak of FM. Inset shows a representative spectral image of fluorescence emission. c) Similar measurements as (b) at the N467C site in the S1–S2 linker as a negative control. d) Percentage change in the TMRM/FM intensity ratio measured from fluorophores labeled at C622, N467C and in blank cells expressing no TRPV1 channel, respectively. *, *P* < 0.05. *n* = 3–8. All statistics are given as mean ± s.e.m. e) Left, a schematic diagram illustrating depolarization‐induced collective movements of charged residues and dipoles in the outer pore region. Right, comparison of TRPV1 structure in the closed state (gray) and the activated state model (blue). Helices are presented as pipes. Conformational changes in charged residues and helixes underlie the unorthodox voltage‐sensing mechanism in TRPV1. f) Locations of outer pore charged residues in the structurally aligned resting and activated state models. The locations of negative charges are approximated by the oxygen atoms in the sidechain (shown as a large sphere). The dashed arrow in blue indicates the central axis through the pore (also defined as the z axis of the models). g) The surface electrostatic potential of the resting and activated states. Positive and negative potentials (in kT/e) are colored in blue and red, respectively.

The TRPV1 closed‐state structure and vanilloids/toxin‐activated structures have been resolved by cryo‐EM.^[^ ^28^, ^29^, ^51^ ^]^ However, these structures are missing a part of the outer pore important for voltage and proton activation; no voltage/proton‐induced open structure is currently available. To understand the structural basis for voltage/proton‐dependent gating, we first modeled the proton‐induced open state structure using Rosetta structural modeling suite (see Methods and Extended Data Figure S10, Supporting Information). Comparison between the proton‐activated state model and the closed state structure suggested two major conformational changes. First, the selectivity filter region moved away from the central axis to yield an apparent open conformation of this upper gate (**Figure** **4**a, gray to red), similar to DkTx‐induced conformational changes observed in the cryo‐EM studies.^[^ ^29^, ^51^ ^]^ Second, the pore turret regions moved substantially closer to each other upon protonation (Figure 4a, dark gray to cyan), which has been previously observed by fluorescence recordings during heat activation.^[^ ^52^ ^]^ Associated with these structural changes are relocation of multiple charged residues (Figure 4f).

To functionally verify the predicted outer pore movement, we employed FRET‐based patch fluorometry^[^ ^53^ ^]^ to simultaneously record conformational and functional states of TRPV1 channels.^[^ ^54^ ^]^ Our structural models predicted that the distance between C622 residues of neighboring subunits (measured at C_*β*_ atoms; Figure 4a, yellow) is reduced by a large amount (about 13 Å) in proton‐induced activation. This large distance change would be readily detectable using fluorescein maleimide (FM) and tetramethylrhodamine maleimide (TMRM) as a FRET pair.^[^ ^52^ ^]^ Extracellular acidification (pH 5.0) elicited a large current from the fluorophore‐labeled channels (Figure 4b). Fluorescence imaging from the same channel population revealed an increase in the TMRM/FM intensity ratio that indicated an increase in FRET efficiency (Figure 4b). This change in emission spectrum was absent when fluorophores were attached to cysteine introduced at N467 on the S1–S2 linker, or at nonspecific sites on untransfected cells (Figure 4c,d). Therefore, results from the FRET experiments supported the prediction that the turrets move toward each other in the proton‐activated state.

How does the observed outer pore conformational changes produce voltage‐sensitive gating? While much of the detail remains to be elucidated, it is conceivable that relocation of some negatively charged residues within the *V*_m_ field might be the origin of the gating charge (Figure 4e). Indeed, from the activated‐state model, we could identify multiple negatively charged residues in the outer pore region that exhibited complex and collective movements (Figure 4f): D602, D647, E652, and D655 moved downward along the *Z* axis, E601 and E649 moved upward (Extended Data Table S2, Supporting Information). Based on the simplified assumption of a uniform *V*_m_ field that spans a 30 Å vertical depth, these charge movements alone would be more than sufficient to produce 1 e_0_ gating charge movement. It was also noticed that the modeled gating rearrangements would lead to changes in the electrostatic potential surface of the outer pore, in which a strong negative potential in the resting state dissipates as the channel transitions into the activated state (Figure 4g). Such conformational changes present a plausible picture of the voltage‐sensitive gating process: it is the collective gating rearrangements of negatively charged residues and the local electric field that produce an unorthodox mechanism of voltage‐sensing in TRPV1.

## 10 Discussion

Our study suggested that voltage‐sensing in TRPV1 originates from conformational changes of the outer pore, where titratable acidic residues collectively contribute to the total charge movement. Because that the *p* *K*a values for the sidechain of negatively charged Glu or Asp are around 4, extracellular acidification to a pH level of 4.0 would only neutralize about half of the charges in these charged residues, reducing the measurable gating charge by about 50%. It was experimentally infeasible to further decrease extracellular pH in patch‐clamp recordings to fully neutralize all the negative charges, though it is most likely that the about 1 e_0_ total gating charge is collectively contributed by titratable acidic residues. Negatively charged residues in the S2 and S3 of Shaker potassium channel^[^ ^21^ ^]^ and BK channel^[^ ^26^ ^]^ also contribute to the total gating charge of these channels. While there are charged residues in the transmembrane domains of TRPV1, we reason that it is less likely these charged residues contribute substantially the total gating charge, because the S1 to S4 domains of TRPV1 lack noticeable activation‐associated movement^[^ ^28^, ^29^, ^51^ ^]^ and the residues critical for proton gating of TRPV1 identified in previous studies^[^ ^48^, ^55^ ^]^ are all clustered in the outer pore region except for V538,^[^ ^56^ ^]^ a neutral residue located in the S1‐S2 linker. Therefore, it is most likely that the total gating charge in TRPV1 is predominantly composed of titratable acidic residues in the outer pore region.

Upon depolarization, the outer pore charged residues appear to undergo complex rearrangements that are directly coupled to opening of the activation gate, most likely the nearby upper gate at the selectivity filter^[^ ^28^, ^29^, ^51^ ^]^ (Figure 4g). The pore turret is seen to move substantially in our model as well as site‐directed fluorescence measurements in our previous studies^[^ ^52^, ^57^ ^]^ and the present study (Figure 4b–d). Details on the nature of pore turret movements remain unclear, as this segment is missing from the available TRPV1 cryo‐EM structures; nonetheless, functional studies of TRPV1 with a turret perturbed by heat^[^ ^52^, ^58^ ^]^ and ligands^[^ ^59^ ^]^ or in different species^[^ ^60^ ^]^ as well as structural studies of the equivalent part of the orthologous TRPV2 suggest this structure moves substantially between the closed and open states.^[^ ^61^ ^]^

The TRPV1 outer pore is a known hot spot for gating modulation of this polymodal receptor. Small cations such as proton, Na^+^ and Mg^2+^/Ba^2+^/Ni^2+^/Gd^3+^, as well as large peptide toxins such as DkTx, RhTx, and BmP01 all bind to this region to exert their strong gating effects.^[^ ^49^ ^]^ Capsaicin activation has been recently found to affect the outer pore.^[^ ^42^ ^]^ Heat also induces large conformational changes of the outer pore.^[^ ^52^ ^]^ Exploiting charged residues and dipole movements in this gating structure would allow *V*_m_ to function essentially as a “master gain setter” to tune the channel's sensitivity to all major stimuli (see Figure 1c), a feature that could be of high practical significance for this nociceptor. For example, under pathological conditions such as bone cancer, TRPV1‐expressing dorsal root ganglion neurons exhibit higher excitability.^[^ ^62^ ^]^ The resting membrane potential of these neurons is depolarized, which leads to elevated nociceptive activities of TRPV1, contributing to the severe and intractable pain in the bone cancer. When TRPV1 activity is modulated by hyaluronan, firing frequency of DRG neurons is also changed.^[^ ^63^ ^]^ In this regard, understanding the voltage‐sensing mechanism may facilitate future pharmaceutical efforts targeting this pain sensor.

Comparing to many noxious stimuli for TRPV1 such as capsaicin and heat, voltage is a relatively mild activator. As we discussed previously,^[^ ^46^ ^]^ the weak voltage sensitivity ensures that TRPV1 does not act as a dedicated *V*_m_ sensor, which is critical for its nociceptive functions. The total charge movement of about 1 e_0_, though only a fraction of that in classic S4‐based voltage‐gated channels, can effectively drive man‐made transistors in conventional electronic devices^[^ ^5^ ^]^ and many biological functions of membrane proteins. As pointed out by Hodgkin,^[^ ^64^ ^]^ gating charge movement absorbs energy from electrical signaling and acts as a load of the system, therefore evolution tends to optimize its utility. We showed recently that weak voltage dependence is a crucial feature that allows TRPV1 to respond to diverse noxious stimuli including heat, whereas the high voltage sensitivity of Kv channels effectively prohibits the channel's intrinsic heat sensitivity.^[^ ^46^ ^]^ It is plausible that similar trade‐offs can be found in other voltage‐regulated polymodal TRP channels.

When the S4 of Kv channels is functionally decoupled from the pore by introduced mutations, a weakly voltage‐dependent (*q* of ≈ 1.8 e_0_), concerted transition is revealed.^[^ ^65^ ^]^ In CNG channels, S4 (containing multiple charged residues) appears to be naturally decoupled from the gate,^[^ ^66^ ^]^ and the channel's intrinsic weak voltage dependence (*q* of ≈ 0.74 e_0_) likely comes from outer pore movement.^[^ ^67^ ^]^ Therefore, it is likely that the outer pore‐mediated voltage‐sensing mechanism is broadly used in ion channels. Indeed, many TRP channels exhibit similar shallow voltage dependence, sparse charged residues in S4 but richly distributed charged residues in the outer pore, a region seen to under tremendous evolutional selection.^[^ ^68^ ^]^ While activation of these channels is also critically regulated by *V*_m_, their voltage‐sensing mechanism is unknown (Figure 1d, section in red). The unorthodox voltage‐sensing mechanism we identify in TRPV1 may help elucidate the function of these channels and other membrane proteins.

## Conflict of Interest

The authors declare no conflict of interest.

## Author Contributions

F.Y. and J.Z. conceived the project; F.Y., X.L.Z., B.H.L., and X.X. conducted the experiments including patch‐clamp recording, mutagenesis, molecular modeling and data analysis. V.Y.‐Y. supervised molecular modeling and revised the manuscript. J.Z. and F.Y. prepared the manuscript. J.Z., V.Y.‐Y., and F.Y. supervised the project, participated in data analysis and manuscript writing.

## Supporting information

Supporting Information

## Data Availability

All data needed to evaluate the conclusions in the paper are present in the paper and/or the Supporting Information. Additional data available from authors upon request.
