# Supplementary material for: An Unorthodox Mechanism Underlying Voltage Sensitivity of TRPV1 Ion Channel
Source: Adv Sci (Weinh). 2020 Sep 21;7(20):2000575. doi: 10.1002/advs.202000575 (PMC7578911; doi:10.1002/advs.202000575)
Supplement: Supplementary file 1 — Supporting Information [file ADVS-7-2000575-s001.pdf]

WILEY-VCH

© 2020 Wiley-VCH GmbH

ADVANCED SCIENCE
Open Access

Supporting Information

for *Adv. Sci.*, DOI: 10.1002/advs.202000575

An Unorthodox Mechanism Underlying Voltage Sensitivity of TRPV1 Ion Channel

Fan Yang, Lizhen Xu, Bo Hyun Lee, Xian Xiao, Vladimir Yarov-Yarovoy, and Jie Zheng**

© 2020 Wiley-VCH GmbH

Supporting Information

An unorthodox mechanism underlying voltage sensitivity of TRPV1 ion channel

Fan Yang[1,2¶], Lizhen Xu[1], Bo Hyun Lee[2], Xian Xiao[2,3], Vladimir Yarov-Yarovoy[2] and Jie Zheng[2¶]

[1] Department of Biophysics, and Kidney Disease Center of the First Affiliated Hospital, Zhejiang University School of Medicine, Hangzhou 310058, China. [2]Department of Physiology and Membrane Biology, University of California, Davis, California 95616, USA. [3]Institute for Basic Medical Sciences, Westlake Institute for Advanced Study, Westlake University, Shilongshan Road No. 18, Xihu District, Hangzhou 310064, Zhejiang Province, China.

[¶] Correspondence and requests for materials should be addressed to F.Y. (email: fanyanga@zju.edu.cn) or J.Z. (email: jzheng@ucdavis.edu)

MATERIALS AND METHODS

Molecular biology and cell transfection. cDNA of Murine TRPV1 (a gift from Dr. Michael X. Zhu, University of Texas Health Science Center at Houston) and Kv2.1 (a gift from Dr. Jon Sack, University of California, Davis) were used in this study. To facilitate identification of channel-expressing cells, eYFP and GFP were fused to the C-terminus of TRPV1 and the N-terminus of Kv2.1, respectively. Tagging of these fluorescence proteins did not alter channel functions, as previously reported[1, 2]. QuickChange II mutagenesis kit (Agilent Technologies) was used to generate point mutations. All mutations were confirmed by sequencing.

HEK293T cells, purchased from and authenticated by American Type Culture Collection (ATCC), were cultured at 37°C with 5% CO_2 in a Dulbecco's modified eagle medium with 10% fetal bovine serum and 20 mM L-glutamine. Transient transfection with cDNA constructs was done with Lipofectamine 2000 (Life technologies) following manufacturer’s protocol. Patch-clamp recordings were performed 1-2 days after transfection.

Chemicals. All chemicals such as capsaicin were purchased from Sigma-Aldrich.

Electrophysiology. Patch-clamp recordings were performed with a HEKA EPC10 amplifier with PatchMaster software (HEKA) in inside-out or whole-cell configuration. Patch pipettes were prepared from borosilicate glass and fire-polished to resistance of ~4 MΩ. For whole-cell recording, serial resistance was compensated by 60%. A normal solution containing 130 mM NaCl, 10 mM glucose, 0.2 mM EDTA and 3 mM HEPES (pH 7.2) was used in both bath and pipette. For solutions with 6.0 < pH < 7.2, HEPES was used as the pH buffer. For solutions with pH ≤ 6.0, HEPES was replaced by 3 mM 2-(N-morpholino)ethanesulfonic acid (MES). When intracellular Na^+ ions were replaced by Mg^{2+} ions, there was no Mg^{2+} in the extracellular solution. To determine a G-V curve, the membrane potential was first clamped at -80 mV for 10 ms and then switched to another clamping voltage stepping from -210 mV to +210 mV with a 10-mV interval for 500 ms, which was then switched to -80 mV. Current amplitude at the steady state during the last 100 ms of voltage steps was averaged to construct the G-V curve. Current signal was filtered at 2.9 kHz and sampled at 10 kHz.

To apply solutions containing capsaicin or other reagents during patch-clamp recording, a rapid solution changer with a gravity-driven perfusion system was used (RSC-200, Bio-Logic). Each solution was delivered through a separate tube so that there was no mixing of solutions. Pipette tip with a membrane patch was placed directly in front of the perfusion outlet during recording. Each membrane patch was recorded for only once.

Temperature control and monitoring. The bath solution was heated using an SHM-828 eight-line heater driven by a CL-100 temperature controller (Harvard Apparatus). We placed a TA-29 miniature bead thermistor (Harvard Apparatus) about 1 mm from the pipette tip to monitor local temperature change. Temperature readout from the thermistor was fed into an analog input port of the HEKA patch-clamp amplifier and recorded simultaneously with channel current. The speed of temperature change was set at a moderate rate of about 0.3 °C/s to ensure that heat activation reached equilibrium during the course of temperature change and the current was recorded at steady state. When the experimental temperature was not controlled, recordings were conducted at room temperature at 24°C. Temperature variation was less than 1 °C as monitored by a thermometer.

Site-directed fluorescence recordings. Structural changes in the turret and other extracellular regions were monitored with site-directed fluorescence recordings as previously described [3]. Briefly, fluorescein maleimide (FM) and tetramethylrhodamine maleimide (TMRM) were used to irreversibly label a native cysteine C622 in the turret region (after removing another native cysteine by mutation C617A), or an introduced cysteine N467C in the S1-S2 linker region after removing both C617 and C622 with an alanine mutation. Fluorescence resonance energy transfer (FRET) between FM and TMRM was measured from voltage-clamped HEK293 cells imaged with an inverted fluorescence microscope (Nikon TE2000-U) using a 40X oil-immersion objective (NA 1.3). An argon laser (Spectra-Physics) was used to provide the

excitation light, with the exposure time controlled by a Uniblitz shutter synchronized with the camera by the PatchMaster software through HEKA amplifier. Spectral measurements were performed with an Acton SpectraPro 2150i spectrograph in conjunction with a Roper Cascade 128B CCD or an Evolve 512 EMCCD camera. The filter cube (Chroma) contained (excitation, dichroic, emission): Z488/20, z488rdc, HQ500lp, so when spectroscopic images were taken from each cell, the excitation at 488 nm was used. The rates of photobleaching FM and TMRM by the excitation light were quantified separately from fluorophores attached to the channel and used to correct for photobleaching during FRET experiments. Proton-dependent changes in fluorescence intensity were also measured and corrected. FRET was quantified from the enhancement of acceptor fluorescence emission due to energy transfer[1, 4]. FRET measurements were done with cells that were patch-clamped throughout the recording. Proton-induced current potentiation and FRET change were recorded simultaneously.

Molecular Modeling. To model the pore region of TRPV1 under neutral and acidic extracellular pH, membrane-symmetry-loop modeling was performed using the Rosetta molecular modeling suite[5]. First, to model the pore region under neutral pH, coordinates of S1-S5, Pore helix, and S6, were taken from TRPV1 channel structures (PDB ID: 3J5P) and kept rigid during loop modeling and finally relaxed. The loop regions between S1 and S2, S2 and S3, S5 and pore helix (turret), the selectivity filter and its linker to S6 were modeled *de novo* in each subunit of the tetramer. During the first round of modeling, Rosetta's cyclic coordinate descent (CCD) loop relax protocol[6] was used and the top 20 cluster center models were passed to the second round. During the second to third rounds of modeling, kinematic (KIC) loop relax protocol[6, 7] was used and the top 20 cluster center models were passed to the next round. During the rest rounds of modeling, kinematic loop relax protocol was used and the top 20 models by score were passed to the next round. From 10,000 to 20,000 models were generated in each round. Models reported here represent the lowest energy models from the last round of iterative loop relax. The TRPV1 models we generated clearly entered an energy funnel through the ten rounds of loop modeling (Extended Data Fig. 9), suggesting that our final model is located at the bottom of energy well. Top 10 models with lowest energy after the last round of loop modeling exhibited good structural convergence (Extended Data Fig. 9).

To model the pore region under acidic pH, we followed the established protocols in Rosetta[8]. Briefly, we modified the membrane energy function terms[9] as described in these reports. Then we added the following command during the loop modeling:

```
-pH_mode true
```

We set the pH value to be 4 by adding this command:

```
-value_pH 4
```

Rounds of CCD and KIC loop modeling were performed with these commands to obtain the model of TRPV1 pore region under acidic pH.

The quality of models under neutral and acidic pH were rigorously assessed by four independent protein structure analysis and verification methods[10]. The metrics of our models were similar to those derived from cryo-EM and crystallography (Extended Data Table 1).

All the molecular graphics of TRPV1 models were rendered by UCSF Chimera[11] software version 1.11.

Data analysis.

To characterize the steady-state G-V curves, a single-Boltzmann function was used:

$$\frac{\mathrm{G}}{\mathrm{G_{max}}} = \frac{1}{1+\mathrm{e}^{-\frac{q_{app}\cdot \mathrm{F}}{\mathrm{RT}}\left(\mathrm{V}-\mathrm{V}_{1/2}\right)}} \quad (1)$$

where G/Gmax is the normalized conductance, $V_{1/2}$ is the half-activation voltage, q_{app} is the apparent gating charge and F is Faraday's constant, R is gas constant, and T is temperature in Kelvin.

To describe the voltage activation kinetics of Kv 2.1 channels, the following equation was used:

$$P_o = \left[\left(\frac{\alpha}{\alpha+\beta}\right)\cdot\left(1-e^{-(\alpha+\beta)\cdot t}\right)\right]^n \quad (2)$$

where α and β are microscopic forward and backward transition rates, respectively, n is the number of independent transitions the channel must transverse before opening. For Kv 2.1 channels we set n to be 4.

To estimate the total gating charge from the voltage dependence of channel activation, we followed the method employed in the study of BK channels[12]. Briefly, the mean activation charge displacement Q_a was first calculated by

$$Q_a = \left(\frac{RT}{F}\right)\cdot\frac{d(\ln P_0)}{dV} \quad (3)$$

We determined the d(lnPo)/dv between two adjacent points in the Ln(Po)-V relation.

Since Qa can only approximate the total gating charge when Po is very small, the estimation of gating charge can be influenced by the value of Po. At acidid pH, Po is increased. So we estimated q using a Q_a/(1-Po) correction for acidic pH measurements[12]. This correction was not applied at neutral pH because of the lower value of Po (Po << 1) at neutral pH.

A smoothing function was imposed on the voltage dependence of Ln(Po):

$$\frac{\mathrm{G}}{\mathrm{G_{max}}} = \frac{1}{1+\mathrm{e}^{-\frac{q_{app}\cdot \mathrm{F}}{\mathrm{RT}}\left(\mathrm{V}-\mathrm{V}_{1/2}\right)}} + base \quad (4)$$

and then the total gating charge was estimated by fitting the foot of the q_a-V curve with a function:

$$\mathrm{Qo} = q_0 + q_1 \cdot \mathrm{B[V]} \quad (5)$$

where B[V] is a Boltzmann function in the form of equation (1), q_0 and q_1 represent the amplitudes of the voltage-independent and voltage-dependent components of Q_o (total gating charge), respectively.

For instance, to estimate the total gating charge in purely voltage activation, we first plotted the log of normalized conductance versus voltage in Fig. 1e (open circles in black). Then this plot was fitted with a smoothing function (equation (4)) (dashed line in red) with the parameters $V_{1/2}$ = 107.99 mV, q_{app} = 0.72 e_0 and base = 0.004.

This smoothing function (dashed line in red in Figure 1e) was then used to calculate q_a according to the equation (3). The q_a from the smoothing function was then plotted versus voltage in Figure 1f as the dashed line in red. Then the foot of q_a(smoothing function)-V curve was fitted with equation (4) to estimate the total gating charge, with the parameters q_0 = 0.002 e_0 and q_1 = 0.928 e_0. The total gating charge was estimated to be 0.93 e_0.

Open probabilities of TRPV1 channel lower than 1% were often difficult to quantify reliably; for this reason, we analyzed voltage dependence at lower open probabilities using NPo from single-channel recordings.

Multi-allosteric gating model. The approach we used for the study of TRPV1 polymodal gating was inspired by the work on Ca^{2+}-sensitive voltage-gated BK channels by Aldrich, Horrigan, and Cui [13]. Similar to these earlier studies, our approach to a general gating model for TRPV1 assumed that the closed-to-open transition, C←→O, and the transition induced by a given activation stimulus, R←→A, are both reversible transitions that are coupled allosterically. The details in our modeling have been described previously[14]. The open probability predicted by this model is calculated by the equation below:

$$Po = \frac{L\cdot(1+Jc\cdot E+Jv\cdot D+Jcv\cdot Jv\cdot D\cdot Jc\cdot E)}{1+D+E+Jcv\cdot D\cdot E+L\cdot(1+Jv\cdot D+ Jcv\cdot Jv\cdot D\cdot Jc\cdot E)} \quad (6)$$

where $D = exp\frac{z\cdot F\cdot(V-V_{half})}{R\cdot T}$, *E* was set to be 100 for saturation concentration of capsaicin. The parameters of this gating model were listed in Extended Data Table 3.

Statistical Analysis. All experiments have been independently repeated for at least three times. The exact number of experiment repeats is provided in figure legends. All statistical data are given as mean ± s.e.m.. Two-tailed Student's *t*-test was applied to examine the statistical significance. N.S. indicates no significance. *, $p < 0.05$; **, $p < 0.01$; ***, $p < 0.001$. Igor Pro version 6.1 (WaveMatrix) and Excel version 2007 (Microsoft) are used for statistical analysis.

EXTENDED DATA FIGURE LEGENDS

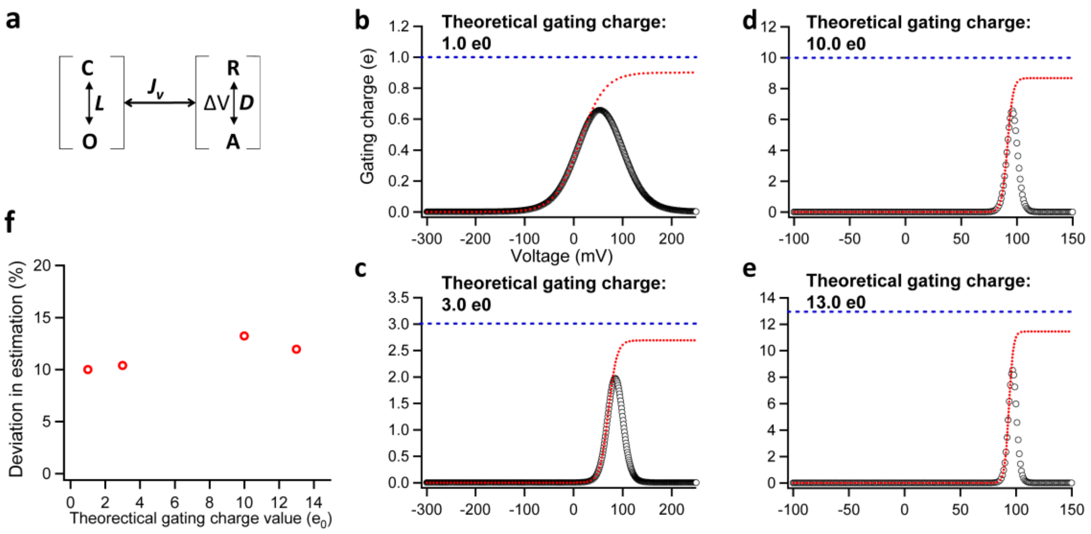

Extended Data Figure 1. Estimation of the deviation between the true value of total gating charge and the measured values. (**a**) The simple allosteric voltage gating scheme employed for the estimation. (**b** to **e**) The voltage dependence of open probability was simulated with the gating scheme in (a) and the theoretical total gating charge was set to 1.0 e_0, 3.0 e_0, 10.0 e_0 and 13.0 e_0, respectively. The simulated voltage dependence of open probability (open circles in black) was further analyzed as described in Method and in Figure 1 (**f**) to derive the voltage dependence of total gating charge (dotted curve in red). (**f**) The measured total gating charge (the maximum value of the dotted curve in red) was compared to the theoretical true values to calculate the deviation in total gating charge estimation.

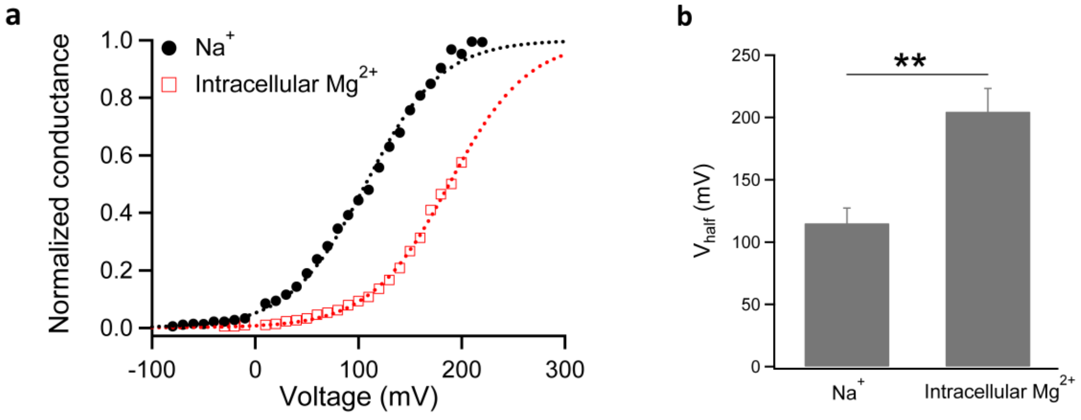

Extended Data Figure 2. Voltage activation of TRPV1 with intracellular Mg^{2+} ions. (**a**) Voltage depence of conductance (G-V) without intracellular Mg^{2+} ions (black) or 100 mM Mg^{2+} ions (red), respectively. G-V curves are fitted to a single-Boltzmann function (dash curves). (**b**) Half activation voltage was significantly increased with intracellular Mg^{2+} ions. *, $P < 0.05$. All statistics are given as mean ± s.e.m.

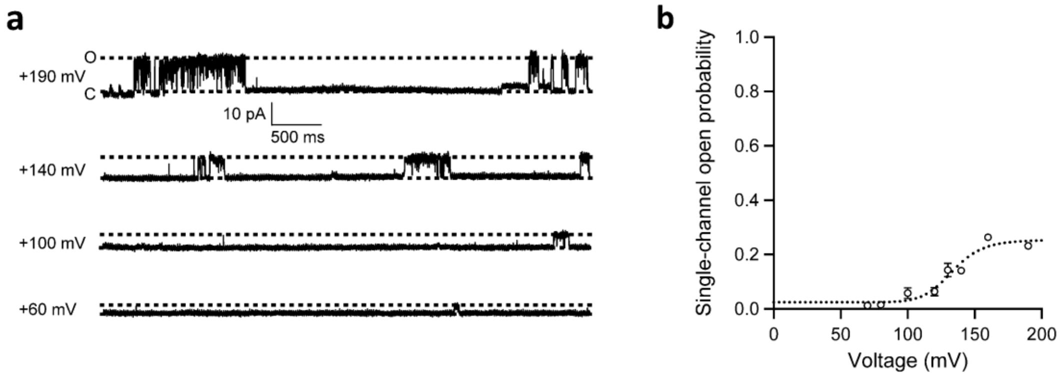

Extended Data Figure 3. Voltage dependence of single-channel open probability. (**a**) Representative single-channel recordings of TRPV1 clamped at different votlages. After voltage activation recording, the membrane patch was perfused with saturation concentration (10 µM) of capsaicin to ensure only one channel was present in the membrane patch. (**b**) Voltage dependence of single-channel open probability fitted to a single-Boltzmann function (dash curves). n = 3. All statistics are given as mean ± s.e.m.

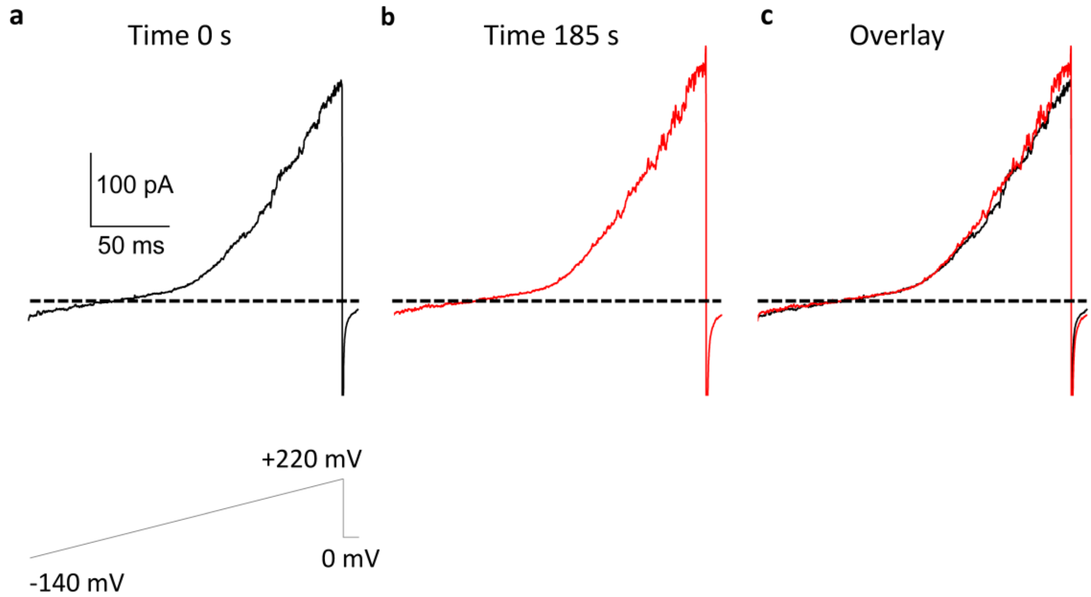

Extended Data Figure 4. The voltage activation of TRPV1 does not diminish upon washing off intracellular factors. (**a**) A representative inside-out patch recording of TRPV1 activated by depolarization during a voltage ramp. This patch was under constant perfusion of bath solution on its exposed intracellular side. (**b**) After constant wash-off for more than three minutes, the patch exhibited a similar current response to the same voltage ramp as in (**a**). (**c**) The overlay of voltage-activated current traces at time 0 s and 185 s demonstrates that washing off intracellular factors did not alter voltage sensitivity in TRPV1. All experiments were performed at 22°C. Only transmembrane voltage was used to activate TRPV1.

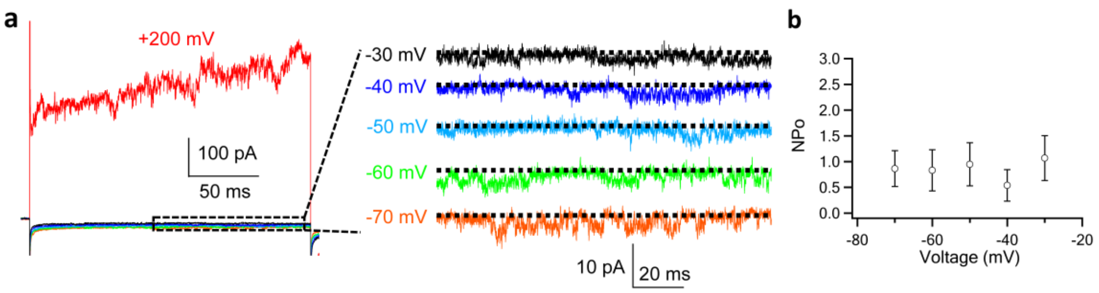

Extended Data Figure 5. Patch-clamp recording of TRPV1 at hyperpolarized voltages. (**a**) A representative inside-out patch recording with voltage stepped from -70 mV to +200 mV. Single-channel activities were clearly discernable at hyperpolarized voltages. The open probability did not further decrease with deeper hyperpolarization beyond -30 mV. (**b**) Voltage dependence of NPo beyond -30 mV remained at a stable level (n = 4).

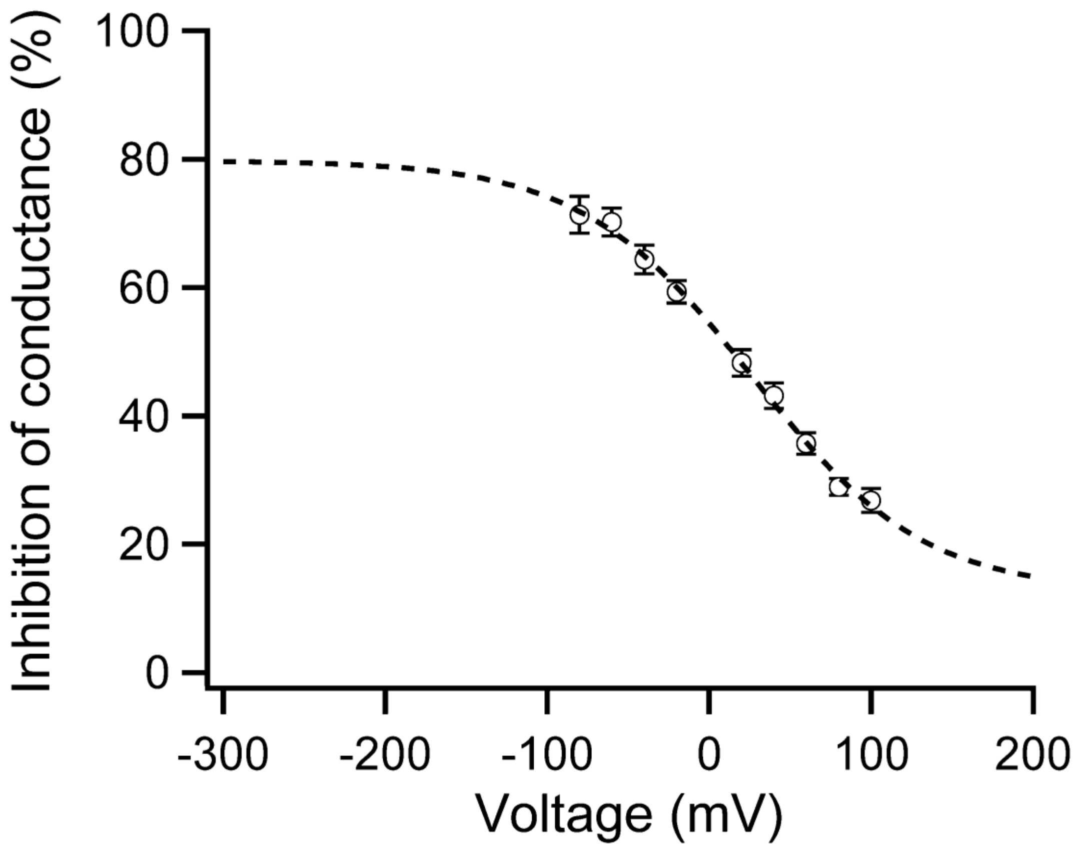

Extended Data Figure 6. Voltage-dependence of proton block of TRPV1 current. This figure is adapted from our previous study[15]. Proton block is calculated from the OFF response recorded in outside-out patches. A Boltzmann function was superimposed ($V_{1/2}$ = 26.6 mV, q_{app} = 0.48 e_0, n = 5-to-9). All statistics are given as mean ± s.e.m.

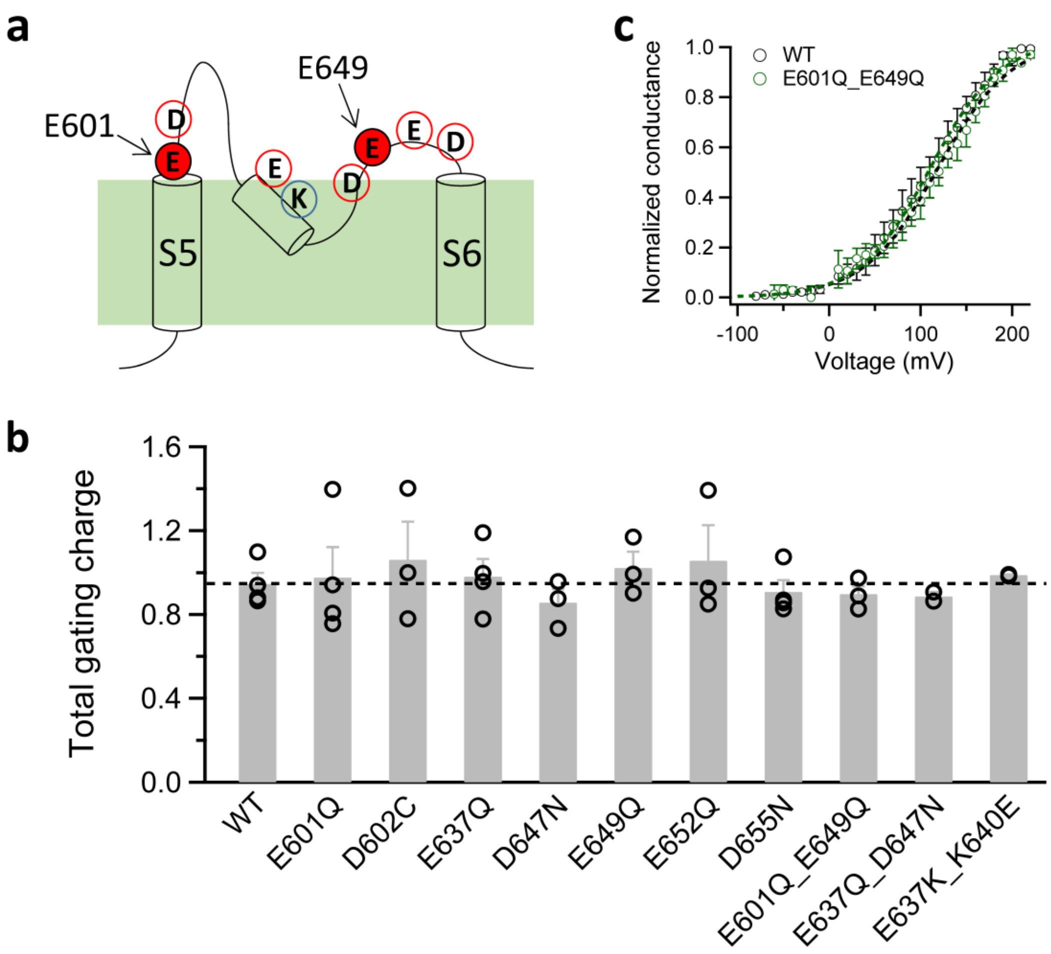

Extended Data Figure 7. Neutralizing individual charged residues in the outer pore near transmembrane voltage field did not eliminate voltage activation in TRPV1. (**a**) A topology plot of the pore region of TRPV1 showing the charged residues near transmembrane voltage field. Two residues, E601 and E649, known to be critical for proton activation are highlighted in red. (**b**) Total gating charge measured from each single and double mutant. A dashed line in black indicate the total gating charge of WT. All data are given as mean ± s.e.m. (n = 3-to-4). (**c**) The E601Q_E649Q double mutant (green) exhibits a similar G-V curve ($V_{1/2}$ = 114.1 ± 10.9 mV, q_{app} = 0.90 ± 0.04 e_0, n = 3) as WT (black).

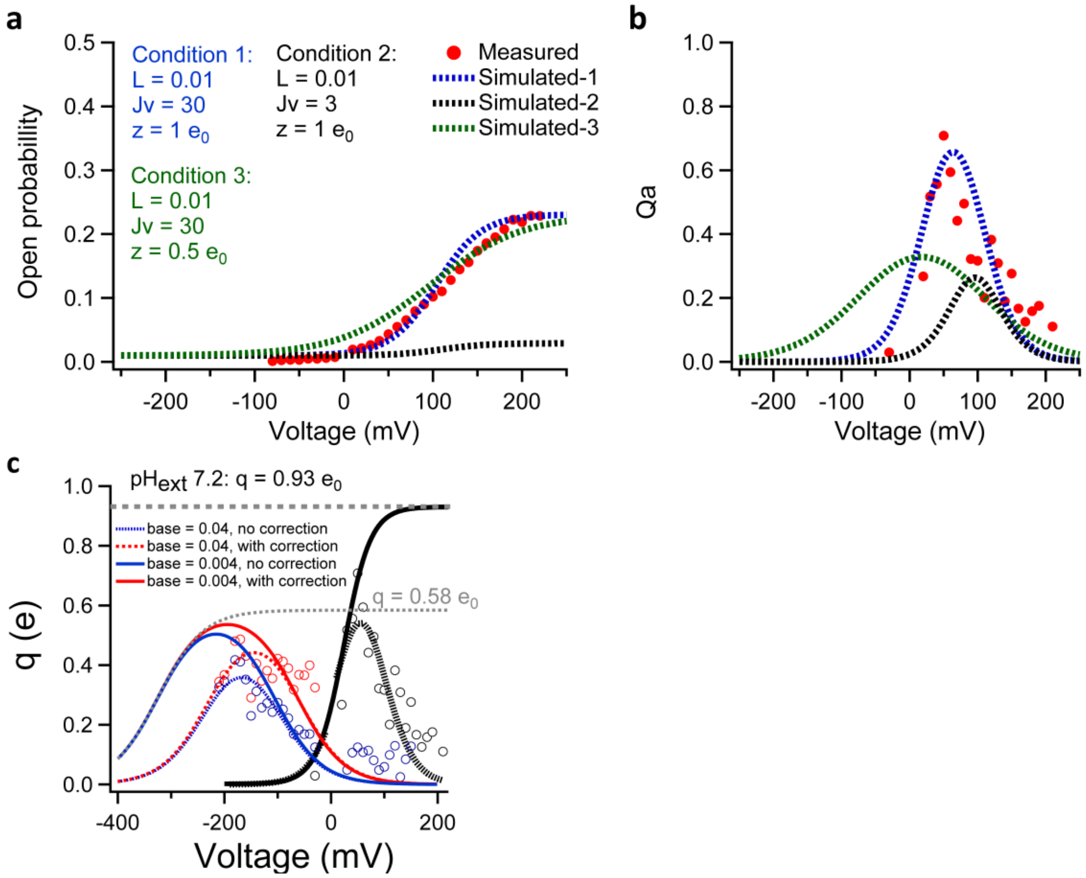

Extended Data Figure 8. Robustness of the gating charge estimation. (**a** and **b**) For a simple allosteric model with voltage activation alone (Extended Data Fig. 1a), reducing the coupling strength between voltage sensor and the gate (Jv) can reduce Qa, but it will largely reduced the maximum open probability. (**c**) 10-fold reduction in the base term in the smoothing function for Qa estimation leads to obvisous deviation of simulated Qa-V curve (solid lines) from experimentally measured values (open circles).

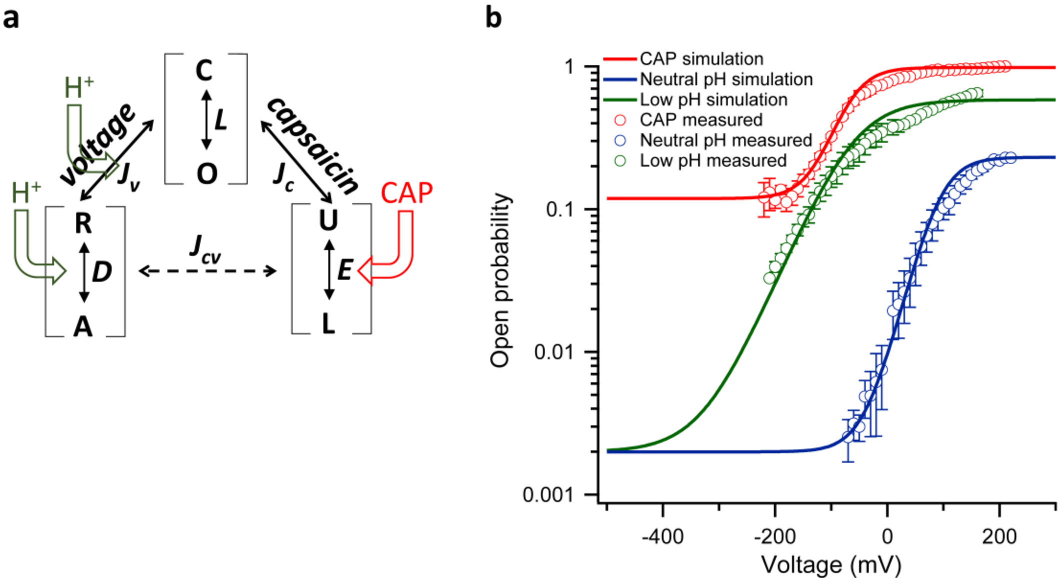

Extended Data Figure 9. A model of multi-allosteric coupling satisfactorily predicts the behavior of TRPV1 gating by voltage (black), capsaicin (blue) and proton (red). In this model (**a**), the voltage-dependent transition R↔A and its coupling to the gate are assumed to be affected by proton, as shown in Scheme III (Figure 3i). Values for the parameters in the model that yielded the voltage-dependent Po shown in (**b**) were listed in Extended Data Table 3.

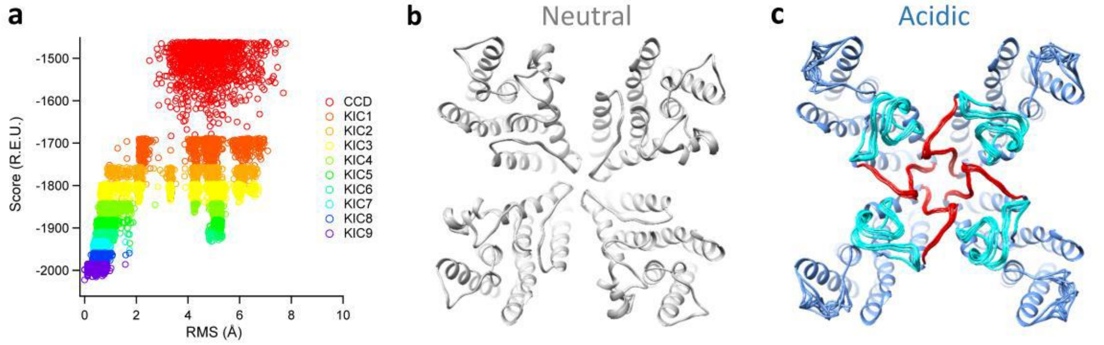

Extended Data Figure 10. Modeling of the outer pore region of TRPV1 under neutral and acidic pH conditions. (**a**) Plot of the Rosetta full-atom score versus *de novo* loop RMS for ten rounds of iterative loop modeling under neutral condition. Low energy models have a more negative value of the full-atom score. (**b**) Overlap of the top 10 models with the lowest energy, which are well converged after iterative loop modeling under neutral condition. (**c**) Top 10 models with the lowest energy are well converged after iterative loop modeling under acidic (pH 4.0) condition. The turret and the linker between pore helix and S6 are colored in cyan and red, respectively.

REFERENCES

[1] W. Cheng, F. Yang, C. L. Takanishi, J. Zheng, J Gen Physiol **2007**, 129, 191.
[2] D. E. Antonucci, S. T. Lim, S. Vassanelli, J. S. Trimmer, Neuroscience **2001**, 108, 69.
[3] F. Yang, Y. Cui, K. Wang, J. Zheng, Proc Natl Acad Sci U S A **2010**, 107, 7083.
[4] J. Zheng, M. C. Trudeau, W. N. Zagotta, Neuron **2002**, 36, 891.
[5] A. Leaver-Fay, M. Tyka, S. M. Lewis, O. F. Lange, J. Thompson, R. Jacak, K. Kaufman, P. D. Renfrew, C. A. Smith, W. Sheffler, I. W. Davis, S. Cooper, A. Treuille, D. J. Mandell, F.

	ERRAT	VERIFY_3D	WHAT_CHECK			PROVE
	Overall quality	Compatibility with sequence	Pass	Warning	Error	Outlier atoms

Richter, Y. E. Ban, S. J. Fleishman, J. E. Corn, D. E. Kim, S. Lyskov, M. Berrondo, S. Mentzer, Z. Popovic, J. J. Havranek, J. Karanicolas, R. Das, J. Meiler, T. Kortemme, J. J. Gray, B. Kuhlman, D. Baker, P. Bradley, Methods in enzymology **2011**, 487, 545.
[6] C. Wang, P. Bradley, D. Baker, J Mol Biol **2007**, 373, 503.
[7] B. Qian, S. Raman, R. Das, P. Bradley, A. J. McCoy, R. J. Read, D. Baker, Nature **2007**, 450, 259.
[8] K. P. Kilambi, K. Reddy, J. J. Gray, PLoS Comput Biol **2014**, 10, e1004018; K. P. Kilambi, M. S. Pacella, J. Xu, J. W. Labonte, J. R. Porter, P. Muthu, K. Drew, D. Kuroda, O. Schueler-Furman, R. Bonneau, J. J. Gray, Proteins **2013**, 81, 2201.
[9] P. Barth, B. Wallner, D. Baker, Proc Natl Acad Sci U S A **2009**, 106, 1409.
[10] J. U. Bowie, R. Luthy, D. Eisenberg, Science **1991**, 253, 164; C. Colovos, T. O. Yeates, Protein science : a publication of the Protein Society **1993**, 2, 1511; R. W. Hooft, G. Vriend, C. Sander, E. E. Abola, Nature **1996**, 381, 272; J. Pontius, J. Richelle, S. J. Wodak, J Mol Biol **1996**, 264, 121.
[11] E. F. Pettersen, T. D. Goddard, C. C. Huang, G. S. Couch, D. M. Greenblatt, E. C. Meng, T. E. Ferrin, Journal of computational chemistry **2004**, 25, 1605.
[12] F. T. Horrigan, R. W. Aldrich, J Gen Physiol **2002**, 120, 267.
[13] F. T. Horrigan, J. Cui, R. W. Aldrich, J Gen Physiol **1999**, 114, 277.
[14] X. Cao, L. Ma, F. Yang, K. Wang, J. Zheng, J Gen Physiol **2014**, 143, 75.
[15] B. H. Lee, J. Zheng, J Gen Physiol **2015**, 146, 147.

Extended Data Table 1. Assessments of model quality. Currently available atomic models of TRP channels derived from cryo-EM or crystallography, as well as two models of TRPV1 we computed with the Rosetta suite under neutral and acidic pH conditions (shaded in green), were assessed by four independent protein structure analysis and verification methods (Green: higher in number is better; Orange and Red: lower in number is better).

**TRPV1 (3J5P)**	86.213	67.47%	77	26	6	7.2%
**TRPV1 (5IRZ)**	63.375	35.79%	70	19	6	7.4%
**TRPV2 (5AN8)**	72.011	38.40%	65	18	4	10.2%
**TRPV6 (5IWK)**	76.552	67.35%	87	31	5	5.2%
**TRPA1 (3J9P)**	66.779	42.32%	56	21	6	0%
**TRPP2 (5K47)**	82.380	44.01%	80	16	1	9.1%
**TRPV1 Model: neutral pH**	88.189	31.64%	77	24	4	3.4%
**TRPV1 Model: acidic pH**	96.850	32.03%	80	21	4	4.5%

Extended Data Table 2. Relative movements of the sidechain of charged residues in the outer pore region of TRPV1 parallel to the transmembrane electric field. Average of the changes in distance along the z axis measured at two oxygen atoms at the end of sidechain are reported (positive: upward movement out of the field; negative: downward movement into the field).

	distance change along z axis (Å)
**E601**	-4.14 ± 1.18
**D602**	-1.17 ± 0.49
**E637**	+0.09 ± 0.09
**D647**	-4.43 ± 0.19
**E649**	-3.14 ± 0.47
**E652**	+3.24 ± 0.66
**D655**	-1.85 ± 0.35

Extended Data Table 3. Parameters for the allosteric gating model of TRPV1 channel.

Parameters	Value
**L**	0.002
**z (e_0) (neutral pH)**	0.93
**z (e_0) (low pH)**	0.55
**Jv (neutral pH)**	150
**Jv (low pH)**	700
**V_{half} (mV) (neutral pH)**	100
**V_{half} (mV) (low pH)**	-15
**Jc**	68
**T (K)**	297.15
**Jcv**	25
